# Exploring the diagnostic potential of immunoglobulin A‐microbiota interplay in liver cirrhosis and spontaneous bacterial peritonitis

**DOI:** 10.1002/kjm2.12876

**Published:** 2024-07-18

**Authors:** Liang‐Jie Zhang, Wen‐Qi Huang, Yuan Zhang, You‐Lian Zhou, Hao‐Ming Xu, Chong Zhao, Yu‐Qiang Nie

**Affiliations:** ^1^ The First Affiliated Hospital of Jinan University Jinan University Guangzhou China; ^2^ Departments of Infectious Disease The First Affiliated Hospital of Bengbu Medical College Bengbu China; ^3^ Department of Gastroenterology the Second Affiliated Hospital, School of Medical, South China University of Technology Guangzhou Guangdong China; ^4^ Department of Gastroenterology The Second Affiliated Hospital, School of Medicine and Institute of Gastroenterology, Zhejiang University Hangzhou China; ^5^ Department of Gastroenterology Guangzhou Digestive Disease Center, Guangzhou First People's Hospital Guangzhou China

**Keywords:** Child–Pugh score, IgA, liver cancer, liver cirrhosis, microbiota

## Abstract

The human gut microbiota significantly impacts health, including liver conditions like liver cirrhosis (LC) and spontaneous bacterial peritonitis (SBP). Immunoglobulin A (IgA) plays a central role in maintaining gut microbial balance. Understanding IgA's interplay with gut microbiota and liver health is crucial. This study explores the relationship between fecal IgA levels, gut microbiota, and liver injury severity. A total of 69 LC patients and 30 healthy controls were studied. Fecal IgA levels were measured using ELISA, and IgA‐coated bacteria were quantified via flow cytometry. Microbiota diversity and composition were assessed through 16S rRNA sequencing. Liver injury severity was graded using the Child–Pugh score. Statistical analyses determined correlations. LC patients had higher fecal IgA levels than controls, correlating positively with liver injury severity. Microbiota diversity decreased with severity, accompanied by shifts in composition favoring pro‐inflammatory species. *Ralstonia* abundance positively correlated with liver injury, whereas *Faecalibacterium* showed a negative correlation. Specific microbial markers for SBP were identified. Functional profiling revealed altered microbial functionalities in LC and SBP. Elevated fecal IgA levels, coupled with microbiota alterations, correlate with liver injury severity in LC patients. Modulating gut microbiota could be a promising strategy for managing liver‐related conditions. Further research is needed to understand underlying mechanisms and translate findings into clinical practice, potentially improving patient outcomes.

**Abbreviations**
EDTA: ethylenediaminetetraacetic acid
EP: Eppendorf
FMT: Fecal microbiota transplantation
HC: healthy control
HSP: Henoch–Schönlein purpura
LC: liver cirrhosis
LEfSe: Linear discriminant analysis effect size
MELD: model for end‐stage liver disease
N‐SBP: nonspontaneous bacterial peritonitis
PBS: phosphate‐buffered saline
PCoA: Principal co‐ordinates analysis
SBP: spontaneous bacterial peritonitis

## 1 INTRODUCTION

The human body is a complex ecosystem, populating with an estimated 10^14^ bacterial cells. This number astonishingly outnumbers human cells by a factor of 10. This vast community of microorganisms, known as the human microbiota, is most densely populated in our gastrointestinal tract.^1^, ^2^ The gut microbiota is primarily characterized by the dominance of two phyla: Anabaena and thick‐walled Bacteria. In contrast, other phyla such as Aspergillus, Micrococcus, Actinobacteria, Clostridium, and Cyanobacteria make up only a small fraction of this microbial diversity.^3^ Research has increasingly underscored the crucial role these microorganisms play in human health and disease. A prime example is the bacterium *Faecalibacterium*, whose reduced levels have been related to various inflammatory conditions, most notably inflammatory bowel disease (IBD).^4^ This bacterium's presence also appears to be diminished in a range of other conditions such as colorectal cancer (CRC), dermatitis, and depression. *Faecalibacterium prausnitzii*, an anaerobic bacterium, is a major component of the intestinal flora and the most significant butyrate‐producing bacterium in the human colon. It is considered a bioindicator of human health, and changes in its population (specifically decreases) have been associated with promoting inflammatory processes. Interestingly, reports have highlighted that the amount of *F. prausnitzii* is negatively correlated with the activity of IBD and colorectal cancer.^5^ This suggests that *F. prausnitzii* could potentially be used as an active ingredient in probiotic preparations to balance gut microbiota, offering a promising therapeutic strategy for IBD and colorectal cancer. Another bacterium, *Ralstonia*, is an aerobic, gram‐negative, nonfermentable rod‐shaped bacillus. It is an opportunistic pathogen that can lead to infections in immunocompromised hosts.^6^ Some studies have reported a positive correlation between the relative abundance of *Ralstonia* and serum uric acid levels, and intestinal *F. prausnitzii* has been associated with significant increases in endotoxin levels and worsening of glucose intolerance in obesity.^7^

The microbiota's influence extends beyond the gut, with its metabolites circulating throughout the body and impacting various organs.^8^, ^9^ Among these organs, the liver, crucial for metabolic processes, is particularly vulnerable to the effects of microbial metabolites. Exposure to certain metabolites can cause liver damage, thereby contributing to conditions such as fibrosis, cirrhosis, and an elevated risk of hepatocellular carcinoma (HCC).^10^, ^11^ This highlights the intricate relationship between gut microbiota and liver health, as disruptions in the balance of gut microorganisms can potentially influence the development and progression of liver diseases, including HCC.^12^, ^13^

Gut microorganisms play a crucial role in health, with disturbances linked to diseases like enteritis, diabetes, hepatitis, and more.^14^, ^15^ These microorganisms help maintain homeostasis by secreting immunoglobulins, especially immunoglobulin A (IgA), which is vital in the intestine for regulating microbes and preventing infection by pathogenic organisms when deficient.^16^

IgA is also key in mucosal immune responses across various body sites, being the most abundant antibody in these areas.^17^ It interacts with bacteria through multiple mechanisms, aiding in expelling pathogens and supporting beneficial bacteria.^18^ An imbalance in intestinal flora is more pronounced in those lacking IgA.^19^

Furthermore, recent research has unveiled potential relations between gut microbiota dysbiosis and HCC pathogenesis.^20^ Studies have demonstrated the impact of microbial metabolites, such as butyrate, on inhibiting HCC development through various cellular mechanisms.^21^ Additionally, disruptions in gut microbiota composition have been observed in HCC patients, suggesting a potential role in hepatocarcinogenesis.^22^ While the direct connections between gut microbiota and the progression from cirrhosis to HCC are still being elucidated, emerging evidence suggests that factors such as immunoglobulin deficiencies may exacerbate the risk of HCC recurrence.^23^, ^24^ Understanding these connections is crucial for developing targeted interventions to mitigate the risk and progression of HCC and other liver‐related conditions.^25^, ^26^

Our study builds upon this foundation by highlighting the significance of IgA in liver cirrhosis (LC) complications, drawing parallels with previous findings linking IgA to conditions like spontaneous bacterial peritonitis (SBP) and Henoch–Schönlein Purpura (HSP). By identifying shifts in IgA‐coated bacteria composition in cirrhosis patients, we observed an increase in pro‐inflammatory species and a decrease in symbiotic bacteria, reflecting the complex relationship between gut microbiota, IgA, and liver health. Furthermore, our findings suggest diagnostic potential in correlating *Ralstonia* prevalence with liver injury severity. High IgA levels were associated with reduced microbial diversity, implicating IgA in gut microbiota modulation and underscoring its potential role in liver health. Notably, the negative correlation between *F. prausnitzii* and liver injury suggests its protective role in cirrhosis. These insights emphasize the need for further investigation to elucidate the clinical implications of the intricate interplay between IgA, gut microbiota, and liver health.

## 2 METHODS

### 2.1 Patients and volunteers

We studied patients with LC who were admitted to the infection department of the First Affiliated Hospital of Bengbu Medical College between June 2021 and December 2022. The HC group comprised relatives of these patients who were found to be healthy on the basis of laboratory tests and detailed physical examinations. The LC group consisted of 69 patients with a mean age of 51.68 ± 10.92 years, comprising 54 men and 15 women. Among these patients, 24 individuals were also diagnosed with HCC. The HC group comprised 30 healthy individuals (age, 48.77 ± 10.97 years; 22 men and 8 women). The age and sex ratios of the two groups were comparable. Controls are abbreviated as HC, LC is abbreviated as cirrhotic patients, the different grades of Child–Pugh score are abbreviated as Child‐Pugh A, B, and C; SBP is abbreviated as SBP; non‐SBP (N‐SBP) is abbreviated as N‐SBP, HCC is abbreviated as Cancer; and nonHCC is abbreviated as N‐Cancer; Basic information and characteristics of the patients were shown in Table 1.

**TABLE 1:** Basic information and characteristics of the patients.

Parameter	HC	Liver cirrhosis	Cancer	SBP
Number	30	26	24	19
Age	48.77 ± 10.97	51.68 ± 10.92	51.83 ± 11.50	50.79 ± 10.18
Gender				
Male	22	19	19	16
Female	8	7	5	3
BMI(Kg/m^2^)	21.53 ± 4.96	21.63 ± 4.84	21.46 ± 5.17	21.92 ± 4.77
Child–Pugh	5.00 ± 0.00	8.44 ± 2.44	6.79 ± 2.25	8.66 ± 2.54
Meld	4.11 ± 1.27	8.41 ± 1.75	8.23 ± 1.66	8.81 ± 1.84
ALB(g/L)	48.94 ± 4.95	34.11 ± 3.98	34.68 ± 3.53	32.47 ± 3.79
TBIL (μ mol/L)	17.74 ± 3.31	27.04 ± 5.74	28.32 ± 7.05	32.58 ± 29.71
AFP (ng/mL)	8.21 ± 4.04	18.87 ± 13.77	1109.25 ± 1082.90	21.96 ± 25.53
Fecal IgA (μg/0.1 g)	8.44 ± 2.06	10.74 ± 3.29	10.69 ± 4.34	12.31 ± 3.75
Hyperlipidemia	2/30		5/69	
Fatty liver	None		None	
Alcohol abuse	None		None	
Diabetes	None		None	

The extent of liver injury in patients with LC was judged using Child–Pugh and model for end‐stage liver disease (MELD) scoring systems. The Child–Pugh scoring system is a method for assessing the severity of cirrhosis based on clinical data of patients with alcoholic cirrhosis, including five indicators of hepatic encephalopathy, ascites, albumin, bilirubin, and PT (Annex 1), and liver function can be classified into A, B, and C according to the patients' scores; whereas the MELD scoring system includes five indicators, including serum bilirubin, creatinine (Scr), INR, and hepatic etiology or serum sodium (Annex 2). Based on the Child–Pugh scoring system, the patients were classified into three groups, namely groups A (Child–Pugh score 5–6), B (Child–Pugh score 7–9), and C (Child–Pugh score 10–15). Considering the dynamic nature of the Child–Pugh score and its susceptibility to acute illnesses like SBP, it is essential to use the score obtained under stable conditions, free from acute illness, to accurately reflect the real status of liver injury. Hence, patients with SBP are excluded from the liver injury comparison. All treatments of patients were approved by the Ethics Committee of The First Affiliated Hospital of Bengbu Medical College (2021‐164).

The diagnostic criteria were based on the Chinese guidelines for the diagnosis and treatment of LC in 2019, Cirrhosis is classified into five stages, compensated (stage 1 and 2) and decompensated (Stage 3, 4, and 5), according to the presence or absence of complications such as ascites, esophageal variceal bleeding and hepatic encephalopathy. Compensated cirrhosis is judged by liver tissue biopsy, liver function (albumin, PTA), blood routine (platelets, white blood cells), LSM (liver stiffness MEASUREMENT) test, imaging, endoscopy, and other comprehensive judgments, whereas compensated cirrhosis is judged by the presence or absence of typical portal hypertension on imaging.^16^ The diagnosis of liver cirrhosis mainly relies on imaging and histology. We excluded patients having^1^ undergone an abdominal surgery in the last 6 months,^2^ a history of taking probiotic drugs in the last; 3 months,^3^ a history of taking antibiotics in the last; 3 months,^4^ challenges with cooperating with the researchers because of special needs, such as cognitive and hearing impairment and mental disorders,^5^ other diseases, such as hypertension and diabetes,^6^ a recent history of diarrhea or food poisoning.

The laboratory reports of aspartate aminotransferase, alkaline phosphatase, albumin, prothrombin time, gamma‐glutamyl transpeptidase, alanine aminotransferase, and serum total bilirubin, were from the Department of Biochemical Laboratory of the hospital. Abdominal B‐ultrasound findings were obtained from the radiology department of the hospital. The hepatic encephalopathy tests were scored by senior doctors with expertise in infectious diseases. This study was approved by the Ethics Committee of the First Affiliated Hospital of Bengbu Medical College (Bengbu, China). All of the patients signed the informed consent form.

### 2.2 Detection of IgA in feces

Considering that the fecal water content differs among patients, the feces were first freeze‐dried using Scientz‐10 N freeze dryer (Ningbo Scientz Biotechnology Co., Ltd., China). For freeze drying, the feces were first refrigerated at −40°C for 1 h, and the vacuum pump was then started for 23 h. Then, 10 mg of feces were taken and placed in a 15 mL centrifuge tube. To this test tube, we added.

A total of 2 mL of 0.01 mol/L phosphate‐buffered saline (PBS) buffer containing soybean trypsin and ethylenediaminetetraacetic acid (EDTA; soybean trypsin, 0.25 mg/mL; 14% EDTA, 2 μL/mL). An ultrasonic cell crusher was used to homogenize the particles before centrifugation at 626 *g* for 10 min at 4°C. Then, 1 mL of supernatant was transferred into a 2 mL Eppendorf (EP) tube and centrifuged again at 14500*g* for 15 min at 4°C. Then, 800 μL of the resultant supernatant was transferred into a new EP tube. The samples were homogenized by sticking the EP tubes horizontally on the vortex and vortexing vigorously for 5 min. The sample was centrifuged at 400*g* for 5 min and the supernatant was filtered through a 70 μm sterile filter (Fisher) and transferred to a new tube. Centrifuge the tube at 8000 *g* for 5 min. Collect the supernatant and determine the amount of IgA by ELISA kit according to the manufacturer's protocol. Read the values using an ELISA enzyme marker (BioTek) and quantify the sample values by comparing them to the kit IgA standard to fit a four‐parameter logistic curve. The operation was carried out according to the ELISA kit instructions. IgA and IgG kits were purchased from Cloud‐Clone Corp. Wuhan (SEA546Hu/CEA544Hu).

### 2.3 Sorting fecal IgA‐coated bacteria by flow cytometry

Stool samples were taken into 2 mL Eppendorf Biopur tubes and placed on ice. A total of 1 mL of PBS (Corning) was added, and the Eppendorf tube was placed horizontally on a vortex and vortexed vigorously for 5 min to homogenize the sample. Samples were centrifuged at 400*g* for 5 min to precipitate large fragments, and the supernatant was filtered through a sterile 70 μm cell filter (Fisher) and transferred to a new 2 mL tube. Samples were centrifuged at 8000*g* for 5 min and the supernatant was removed by aspiration. The bacterial precipitate was resuspended in PBS 0.25% BSA containing SYTOBC for 30 min on ice. A 50 μL bacterial suspension was stained with 50 μL of 2X mAb premix at a final concentration of 10 μg/mL for 20 min on ice. Cells were washed once with 1 mL of PBS 0.25% BSA, centrifuged at 8000*g* for 5 min, and then resuspended in 50 μL of PBS 0.25% BSA and 10% normal goat serum for 10 min on ice. Add 50 μL of 2X goat antihuman IgG biotin (final concentration 1:400, Southern Biotech) and goat anti‐mouse IgA PE (final concentration 1:800, Southern Biotech) and incubate on ice for 20 min. Cells were washed once with 1 mL of PBS 0.25% BSA, centrifuged at 8000*g* for 5 min, and then resuspended in 100 μL of PBS 0.25% BSA with streptavidin‐APC (1:800, Biolegend), and suspended on ice for 20 min. Cells were washed once with 1 mL of 0.25% BSA, centrifuged at 8000*g* for 5 min, and resuspended in PBS 0.25% BSA with DAPI prior to flow cytometry on an LSRI1 flow cytometer (BD).

We analyzed the fecal IgA‐coated bacteria using a previously reported method.^9^ We used the single staining method to prepare separate PE‐conjugated antihuman IgA bacterial solutions for flow cytometry. IgA‐coated bacteria and nonIgA‐coated bacteria were sourced from anti‐IgA‐stained samples. At least 800,000 events of each sample were collected, waiting for further 16S rRNA sequence.

### 2.4 16S rRNA sequencing

#### 2.4.1 Extraction of genome DNA

We used the cetyltrimethylammonium bromide/sodium dodecyl sulfate method for the extraction of total genome DNA from samples. DNA purity and concentration were monitored on 1% agarose gels, use the Nucleic Acid Protein Analyzer to check the concentration of DNA in the samples as well as the optical densities of the samples at 260 and 280 nm, OD 260 and OD 280, to make sure that the OD 260/OD 280 values are between 1.8 and 2.0, which means that the purity of the DNA meets the requirements. DNA was diluted with sterile water to 1 ng/μL.

#### 2.4.2 Amplicon generation

16S rRNA genes of 16S V3‐V4 were amplified using a specific primer (V4:515F‐806R) with the barcode. For all polymerase chain reactions (PCRs), 15 μL of Phusion High‐Fidelity PCR Master Mix was used (New England Biolabs), along with 0.2 μM of reverse and forward primers and ~ 10 ng of template DNA. Thermal cycling comprised initial denaturation at 98°C for 1 min, followed by 30 denaturation cycles at 98°C for 10 s, annealing at 50°C for 30 s, and elongation at 72°C for 30 s. Final extension was performed at 72°C for 5 min.

#### 2.4.3 PCR product quantification and qualification

Herein, 1× loading buffer (containing SYBR green) and PCR products were mixed in the same volume and subjected to electrophoresis on 2% agarose gel for PCR product detection. PCR products were mixed in equidensity ratios and then purified with Qiagen gel extraction kit (Qiagen, Germany).

#### 2.4.4 Library preparation and sequencing

We used the TruSeq® DNA PCR‐Free Sample Preparation Kit (Illumina, USA) according to manufacturer's recommendations to generate sequencing libraries. The library quality was assessed on the Qubit@ 2.0 Fluorometer (Thermo Scientific) and Agilent Bioanalyzer 2100 system. Finally, the library was sequenced on an Illumina NovaSeq platform.

### 2.5 Statistical analysis

All data were expressed as absolute and/or relative frequencies; mean ± SD was presented on graphs. We used the *t*‐test for comparison between two groups; for comparisons among three or more groups, one‐way analysis of variance was used. The correlation was analyzed by Spearman's or Pearson correlation analysis. *p*‐values of <0.05 were considered statistically significant. SPSS 22.0 (SPSS Inc, Chicago, USA) and GraphPad Prism 8.0 (GraphPad, San Diego, CA) were used for all statistical analyses.

## 3 RESULTS

### 3.1 Characteristics of the study population and the distribution of its fecal IgA content

**FIGURE 1:**
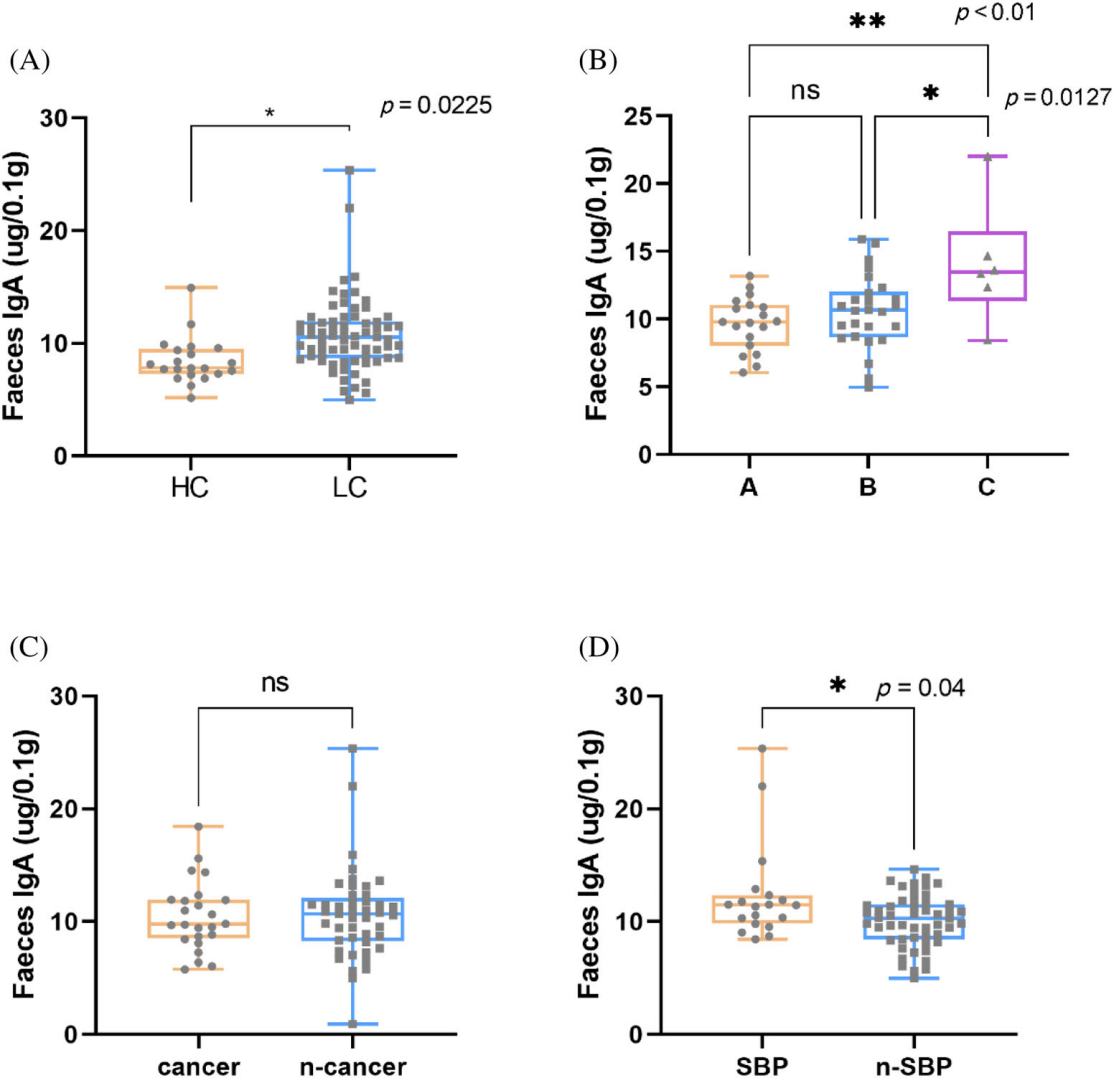
Fecal IgA in different groups. Differences in fecal IgA levels in HC and LC groups (A); Differences in fecal IgA levels in Child–Pugh A, B, and C groups (B); differences in fecal IgA levels in N‐Cancer and Cancer groups (C); differences in fecal IgA levels in N‐SBP and SBP groups (D); *p < 0.05, **p < 0.01, ***p < 0.001, ****p < 0.0001. HC, healthy control; LC, liver cirrhosis; ns, no significance.

**FIGURE 2:**
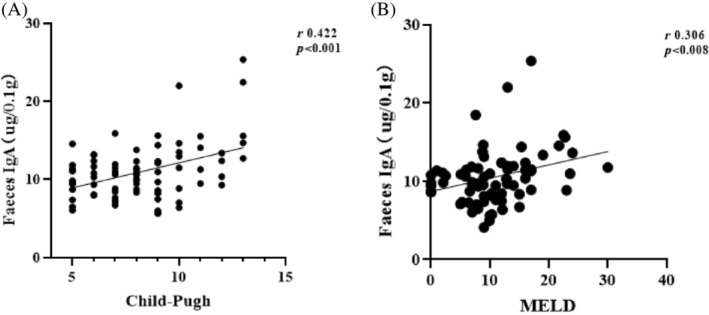
The levels of fecal IgA are related to the Child–Pugh (A) and model for end‐stage liver disease (MELD) scores (B).

Liver tissues of 69 patients with cirrhosis were taken and prepared into pathological sections, 24 of them were found to be HCC patients through pathological examination, whereas the remaining 45 patients' tissues were not found to have cancerous cell features through pathological examination, and stool samples from the family members of 30 patients were also taken as healthy controls (HCs). Analysis of the patients' fecal samples revealed that the IgA content in the feces of patients with cirrhosis was significantly higher than that of the healthy group (*p* < 0.05; Figure 1A), the progression from cirrhosis to HCC also correlate with the IgA content in the patients' feces (Figure 1B). Liver injury in cirrhotic patients was classified according to the Child–Pugh and MELD scoring systems, and it was found that the classification according to Child–Pugh was more detailed than that of MELD, and the results were similar in the two groups, suggesting that there is a correlation between the liver injury of the patients and the fecal IgA content (*r* = 0.422, Child–Pugh; *r* = 0.306, MELD; Figure 2), and that the fecal IgA level rises with the progression of the liver injury in patients with liver pathology, so it is feasible to use patients' fecal IgA levels as a basis for judging the development of chronic liver disease.

### 3.2 Percentage of IgA‐coated bacteria in feces

**FIGURE 3:**
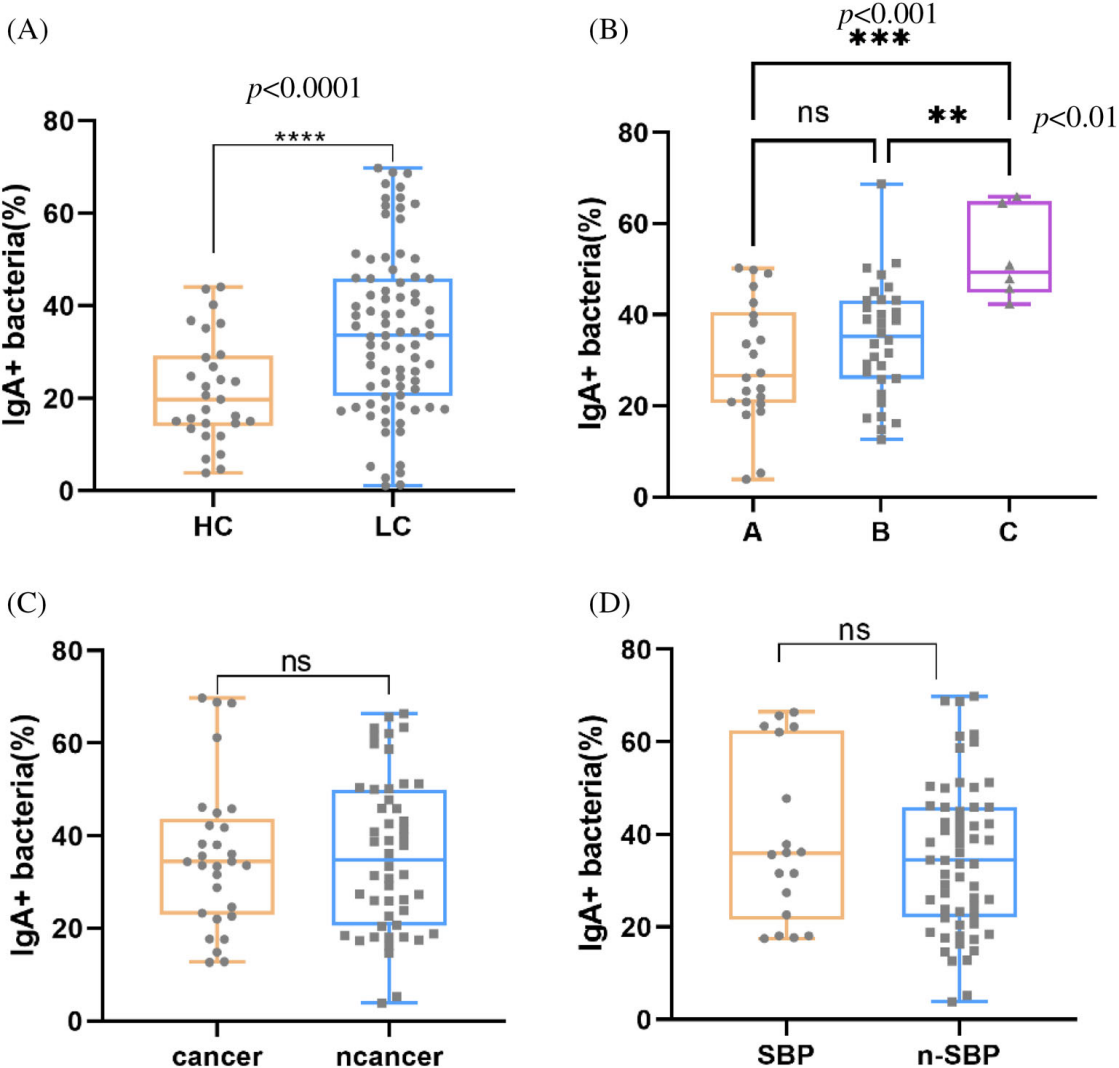
The percentage of IgA‐coated bacteria (IgAP). Differences in fecal IgAP levels in HC and LC groups (A); Differences in fecal IgAP levels in Child–Pugh A, B, and C groups (B); Differences in fecal IgAP levels in N‐Cancer and Cancer groups (C); differences in fecal IgAP levels in N‐SBP and SBP groups (D); *p < 0.05, **p < 0.01, ***p < 0.001, ****p < 0.0001. HC, healthy control; LC, liver cirrhosis; ns, no significance; SBP, spontaneous bacterial peritonitis.

Flow cytometry was employed to quantify the proportions of IgA‐coated bacteria, revealing distinct difference across groups. In the HC group, the average IgA percentage (IgAP) was 21.62% ± 11.42%, whereas in the LC group, it notably increased to 34.48% ± 17.67% (*p* < 0.0001), indicating a significant elevation in IgAP within the LC group compared to the HC cohort (Figure 3A). Within the spectrum of liver disease severity, represented by Child–Pugh grades A, B, and C, IgAP levels exhibited progressive escalation: 30.23% ± 13.39%, 34.42% ± 12.07%, and 42.29 ± 12.51%, respectively. Notably, a significant discrepancy in IgAP levels emerged between groups A/C and B/C in the Child–Pugh classification (*p* < 0.001 and *p* < 0.01, respectively), underscoring a substantial divergence in IgA‐coated bacteria between these categories. However, no statistically significant variations were detected when comparing groups, A/B and B/C (*p* > 0.05), implying a consistent IgAP pattern across these transitions. These findings underscore the utility of the Child–Pugh classification as a more precise indicator of cirrhotic liver injury compared to the MELD classification. Moreover, the positive correlation observed between liver injury severity and the percentage of fecal IgA‐coated bacteria elucidates a potential mechanism underlying disease progression.

Comparing Figures 1 and 3, in HC and LC groups, Child–Pugh's A,B,C groups, Cancer and N‐Cancer groups, it was found that the differences presented in the levels fecal IgA and IgAP; however there was no significant difference (*p* > 0.05) in IgAP between SBP and N‐SBP groups (Figure 3D), However, there was a significant difference (*p* < 0.05) in fecal IgA levels between SBP and N‐SBP groups (Figure 1D).

### 3.3 Differences in microbiological diversity and species difference of fecal flora in patients with different stages of chronic liver disease

**FIGURE 4:**
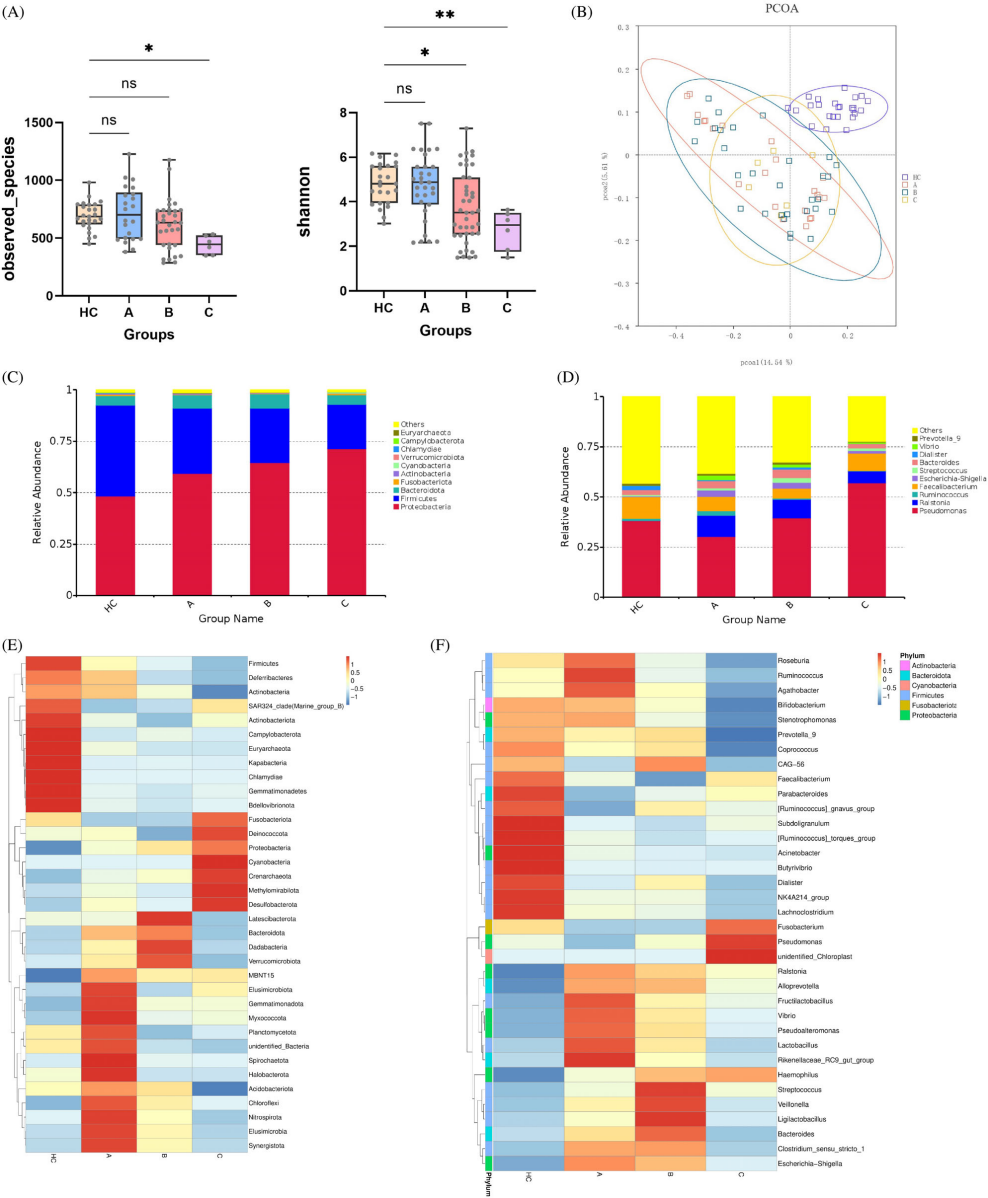
Liver cirrhosis (LC) influence on the microbial diversity and composition. Controls are abbreviated as HC, LC is abbreviated as cirrhotic patients, the different grades of Child–Pugh score are abbreviated as Child–Pugh A, B, and C. (A) α‐diversity in terms of the observed_species and Shannon indexes. (B) The HC, Child–Pugh A, B, and C groups overlapped on the principal co‐ordinates analysis plot, but the Child–Pugh A, B, and C groups showed a trend of separation. LC affected the phylum‐level (C), and genus‐level (D) taxonomic distributions of the feces of the microbial communities. The species abundance heatmap at phylum (E) and genus (F) level.

The Observed Species and Shannon indices are commonly employed metrics for assessing α diversity. The Observed Species index reflects the richness of a group, with higher values indicating greater richness. Conversely, the Shannon index indicates the diversity within a group, with higher values signifying increased diversity. In this study, significant differences were observed in the Observed Species index when comparing the HC group with the Child–Pugh score C groups (*p* < 0.05). And a trend of decreasing median values was evident with increasing Child–Pugh scores. This trend suggests a potential decline in richness as liver disease severity escalates (Figure 4A). In the HC (HC) group, the Shannon index for IgA‐coated bacteria exhibited the highest value at 4.799 ± 0.914, indicative of pronounced diversity. Conversely, individuals with Child–Pugh scores A, B, and C demonstrated decreasing Shannon indices: 4.542 ± 0.4004, 3.98 ± 1.56, and 3.386 ± 1.533, respectively (Figure 4A). Notably, as the Child–Pugh grade escalated, a discernible downward trend in the Shannon index was observed, suggesting a reduction in microbial diversity with increasing liver disease severity. Statistical analysis revealed significant differences in the Shannon index between HC group and grade C (*p* < 0.01, Figure 4A), as well as between the HC group and patients with grade B (*p* < 0.05, Figure 4A). This underscores a notable disparity in microbial diversity between these categories. Furthermore, β diversity analysis of IgA‐coated bacteria using principal co‐ordinates analysis (PCoA) depicted distinct clustering patterns between the HC and LC groups (Figure 4B), suggesting disparate microbial compositions. Additionally, there was a discernible tendency for microbial diversity to diminish with escalating liver injury, as indicated by the clustering patterns observed.

The species difference analyses at the phylum level, IgA‐coated *Proteobacteria* were increased in the LC group (*p* < 0.001), and IgA‐coated *Firmicutes* were decreased in the HC group (*p* < 0.001) (Figure 4C). At the genus level, IgA‐coated bacteria, such as *Ralstonia*, *Streptococcus*, and *Vibrio* (*p* < 0.001, *p* = 0.016, and *p* < 0.001, respectively), were elevated in the fecal flora of the LC group, and IgA‐coated bacteria, such as *Monoglobus*, *Floricoccus*, *Lachnospiraceae*_UCG‐010, and *Mitsuokella* (*p* < 0.001, *p* < 0.001, *p* < 0.001, and *p* = 0.008, respectively), were elevated in the HC group (Figure 4D).

We analyzed the relative bacterial abundance between the HC group and patients with Child–Pugh score A, B, and C at different levels. The following trends of IgA‐coated bacteria were identified with increasing Child–Pugh scores. At the phylum level, *Proteobacteria*, Bacteroidota, Fusobacteriota, and Cyanobacteria increased, whereas *Firmicutes* and Actinobacteria decreased (Figure 4E). At the genus level, *Pseudomonas*, *Ralstonia*, *Escherichia‐Shigella*, *Streptococcus*, and *Vibrio* increased, whereas *Faecalibacterium*, *Dialister*, *Butyrivibrio*, and  *Prevotella_9* showed a declining trend (Figure 4F).

**FIGURE 5:**
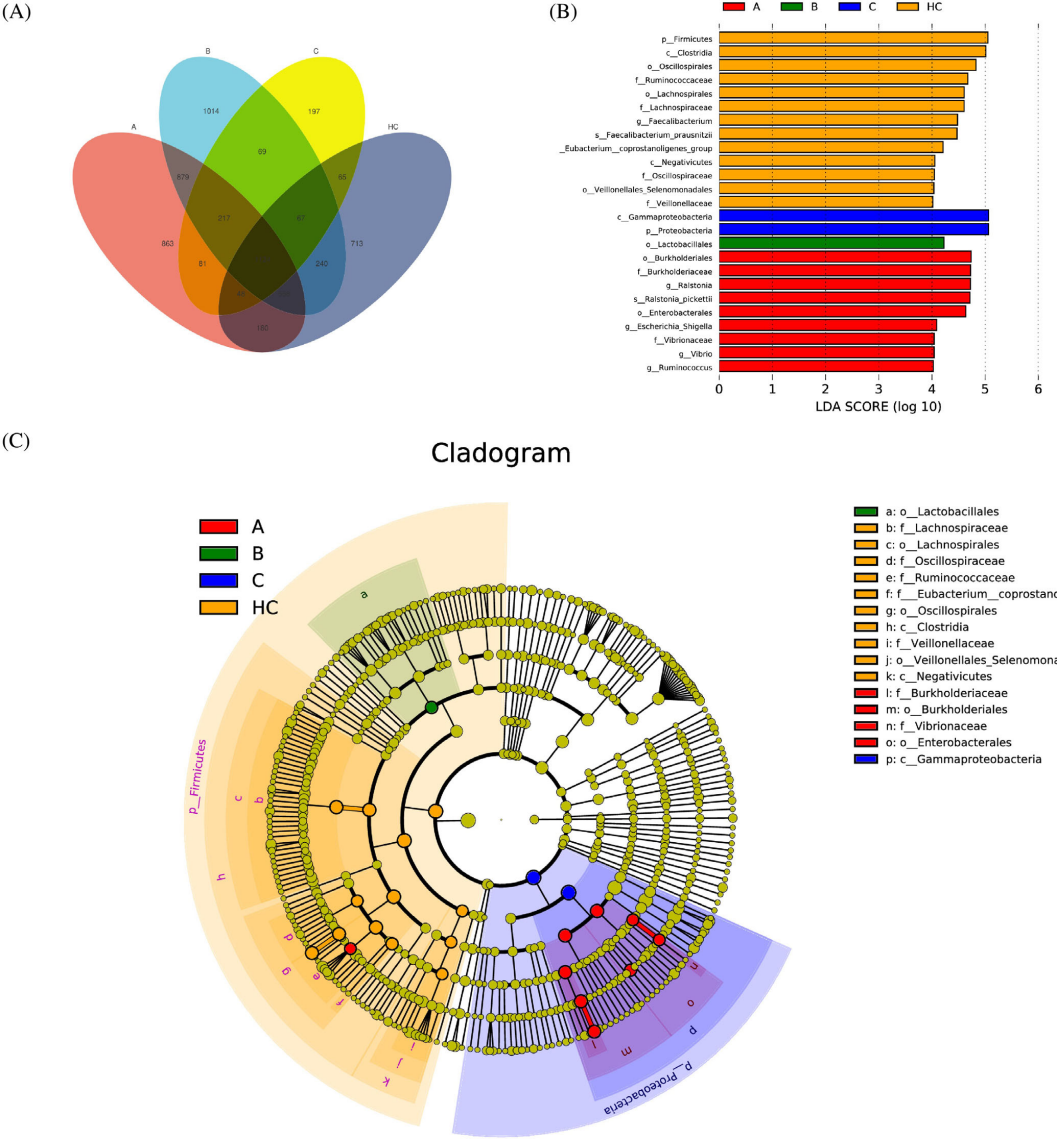
Line discriminant analysis (LDA) effect size analysis. (A) Venn diagram showed the overlapped OTUs of HC, Child–Pugh A, B, and C groups. (B) Linear discriminant analysis effect size between the HC, Child–Pugh A, B, and C groups. (C) Cladogram indicating the phylogenetic distribution of differential gut microbiota between the HC, Child–Pugh A, B, and C groups.

Venn analysis showed Child–Pugh group A had 863 specific operational taxonomic units (OTUs), group B had 1014 specific OTUs, and C with 197 OTUs (Figure 5A). Through the linear discriminant analysis effect size (LEfSe) (LDA = 4) analysis to find the difference marker bacteria between the HC and Child–Pugh group A, B, and C (Figure 5B,C), it can be observed that in the LC, *Ralstonia*, *Ralstonia pockettii* has the potential to become a marker, and at the same time, in the HC, it can be seen that the symbiotic bacteria at various levels including *Faecalibacterium*, *Faecalibacterium prausnitzii*, have the potential to become markers.

### 3.4 Differences in microbiological diversity and species difference of fecal flora in patients with SBP

**FIGURE 6:**
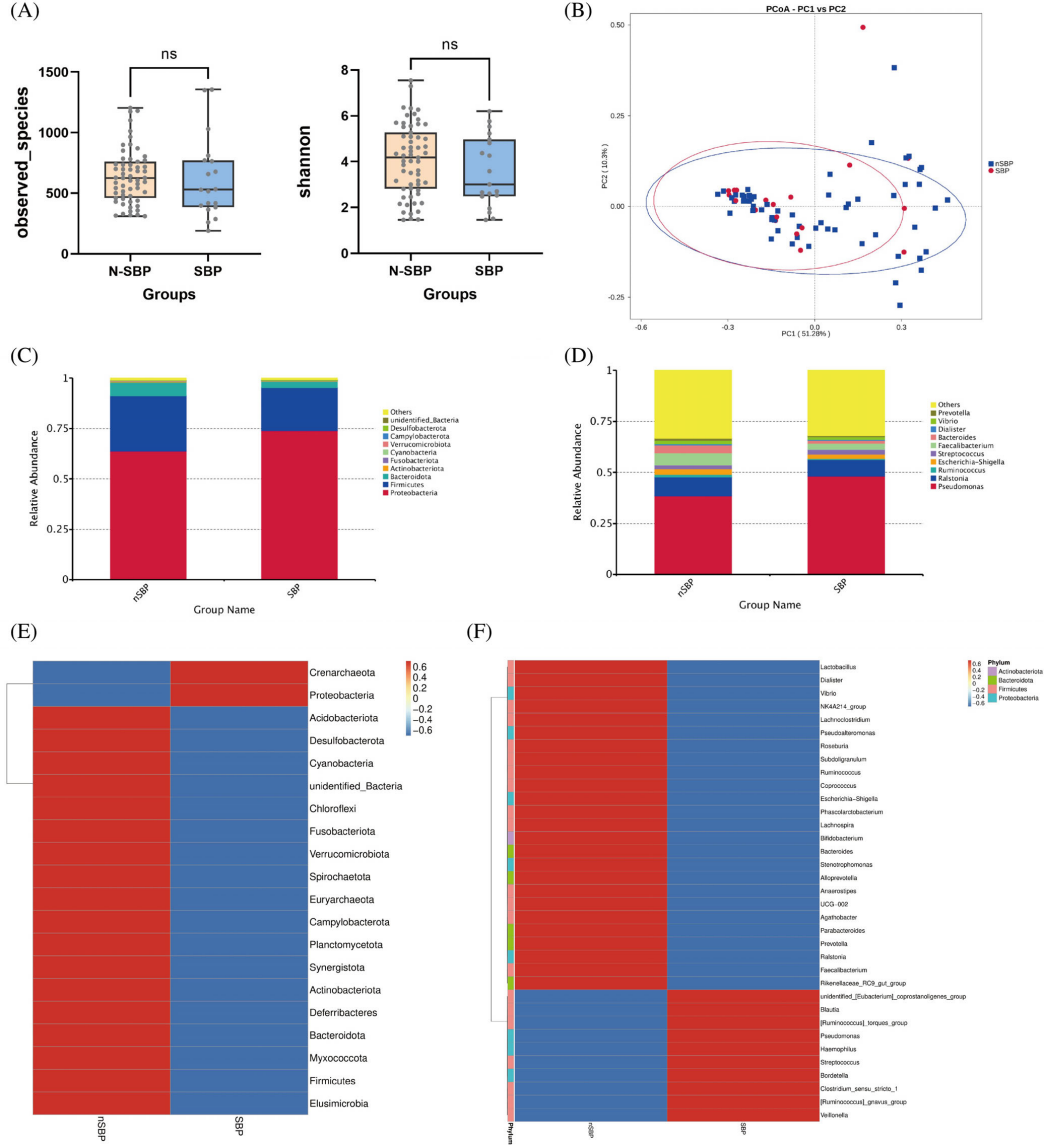
Spontaneous bacterial peritonitis' (SBP) influence on the microbial diversity and composition. SBP is abbreviated as SBP, nonSBP is abbreviated as N‐SBP. (A) α‐diversity in terms of the observed_species and Shannon indexes. (B) The SBP and N‐SBP overlapped on the principal co‐ordinates analysis plot. SBP affected the phylum‐level (C), and genus‐level (D) taxonomic distributions of the feces of the microbial communities. The species abundance heatmap at phylum (E) and genus (F) level.

The Observed Species index reflects the richness of a group, with higher values indicating greater richness. Conversely, the Shannon index indicates the diversity within a group, with higher values signifying increased diversity. In this study, no significant differences were observed in the Observed Species and Shannon index when comparing N‐SBP and SBP group (Figure 6A), suggesting no influence on d microbiological diversity with SBP. Furthermore, β diversity analysis using PCoA depicted no distinct clustering patterns between the HC and LC groups (Figure 6B), suggesting no disparate microbial compositions with SBP.

The species difference analyses at the phylum level, *Proteobacteria* were increased in the SBP group (*p* < 0.05), and Cyanobacteria, *Firmicutes* were decreased in the SBP group (Figure 6C). At the genus level, *Pseudomonas*, *veillonella* (*p* < 0.05, respectively), were increased in the fecal flora of the SBP group, and *Faecalibacterium* was decreased in the SBP group (Figure 6D).

We analyzed the relative bacterial abundance between the N‐SBP and SBP group at different levels. The following trends of IgA‐coated bacteria were identified with SBP. At the phylum level, Proteobacteria, Crenarchaeota, increased, whereas *Firmicutes*, Bacteroidota and Actinobacteria decreased (Figure 6E). At the genus level, *Pseudomonas*, *Veillonella*, *Streptococcusm*, *[Ruminococcus]_gnavus_group*, *Clostridium_sensu_stricto_1*, *and Haemophilus* increased, whereas *Faecalibacterium*, *Dialister*, *Roseburia*, *Prevotella*, and *Bacteroides* showed a declining trend (Figure 6F).

**FIGURE 7:**
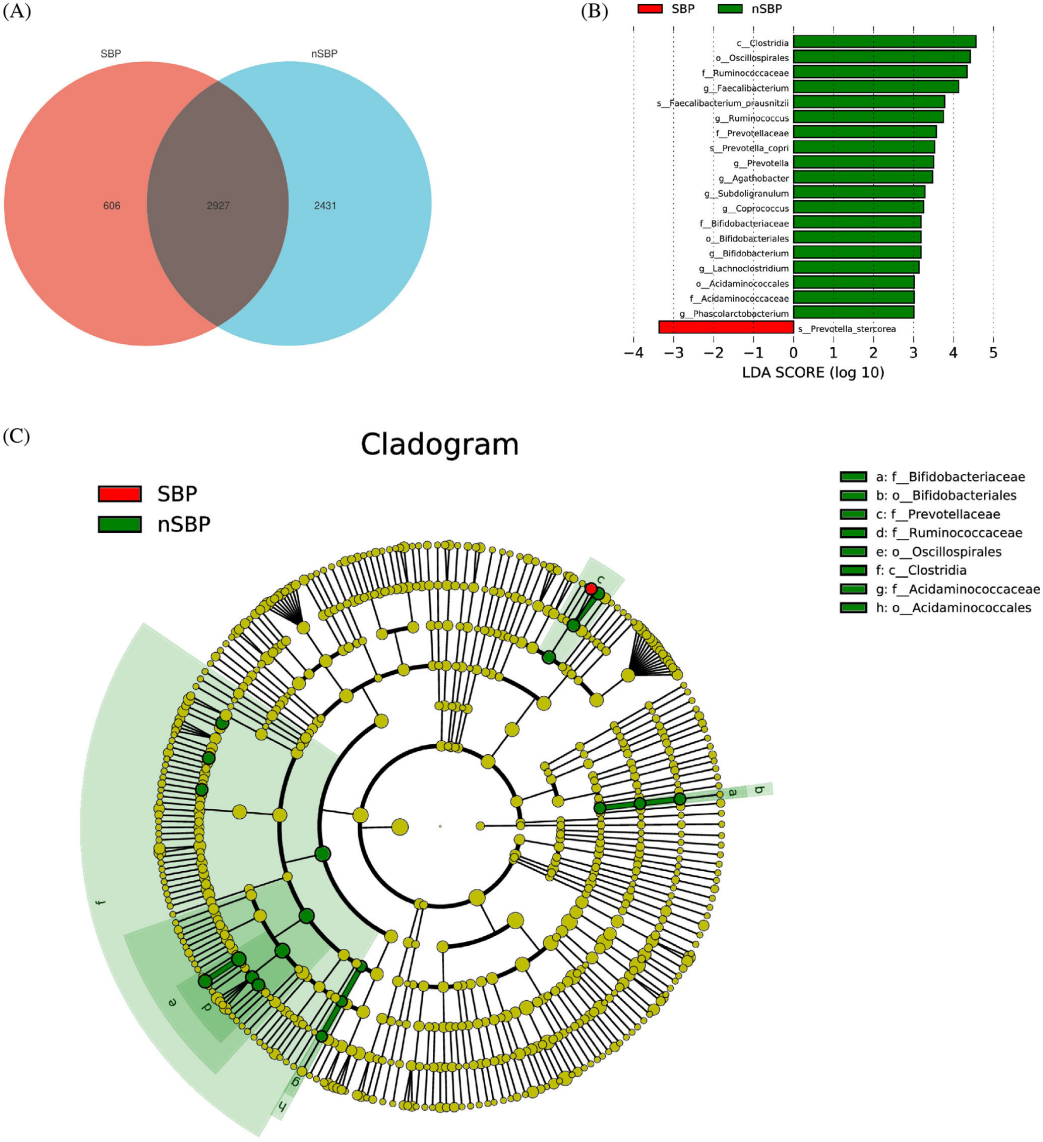
Line discriminant analysis (LDA) effect size analysis. (A) Venn Diagram showed the overlapped OTUs of spontaneous bacterial peritonitis (SBP) and N‐SBP. (B) Linear discriminant analysis effect size between the SBP and N‐SBP. (C) Cladogram indicating the phylogenetic distribution of differential gut microbiota between the SBP and N‐SBP.

Venn analysis showed SBP group had 606 specific OTUs (Figure 7A). Through the LEfSe (LDA = 3) analysis to find the difference marker bacteria between the N‐SBP and SBP group (Figure 7B, C), it can be observed that in the SBP, *Prevatella stercorea*, *Ruminococcus*, *Faecalibacterium*, *Faecalibacterium prausnitzii*, and *Prevatella copri* have the potential to become markers.

### 3.5 Functional profiling of microbiota in LC and SBP

**FIGURE 8:**
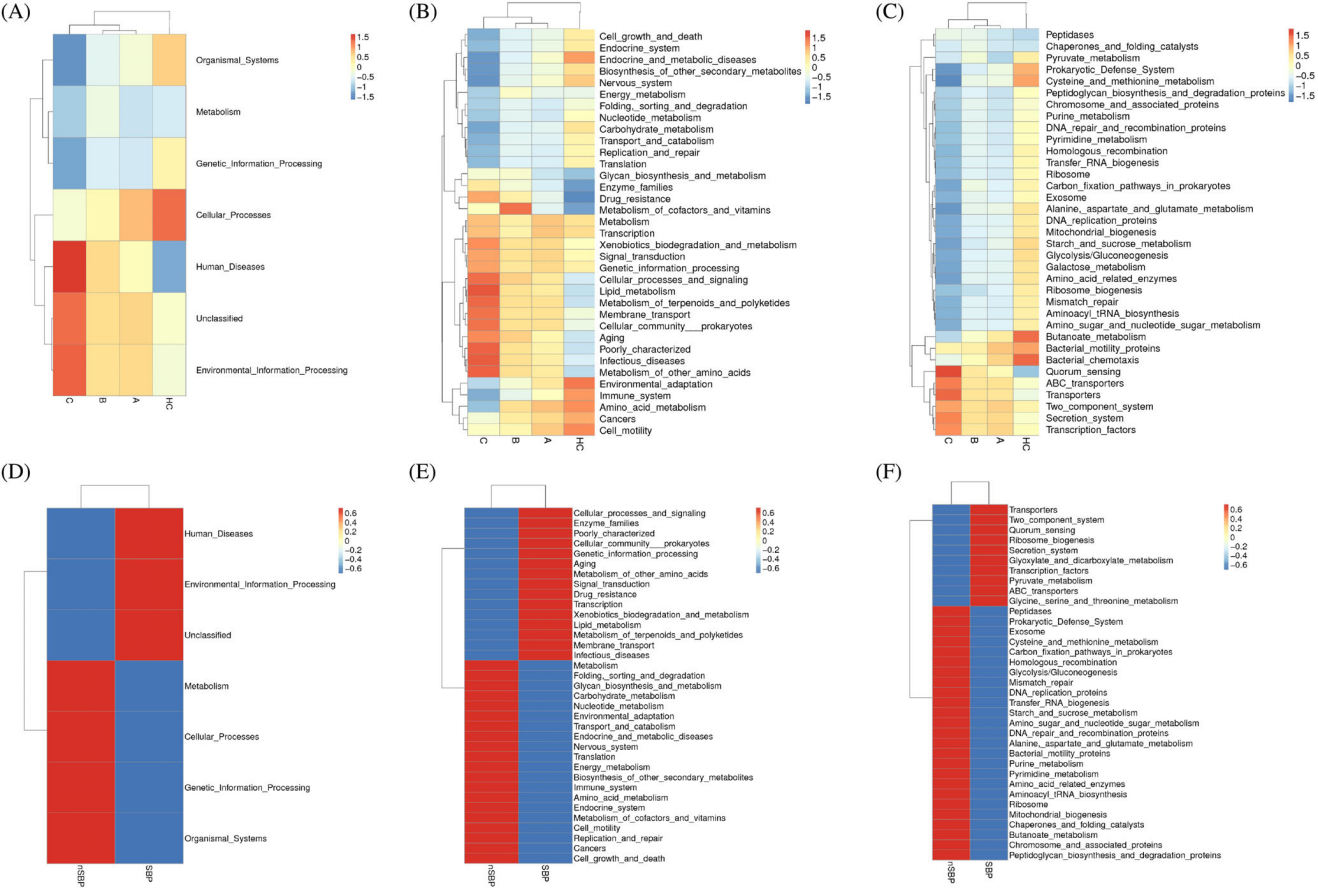
The effect of liver cirrhosis and spontaneous bacterial peritonitis on microbial function. Correlation analysis of differential gut microbiota and differential metabolites between HC, Child–Pugh A, B, and C groups in (A) LV1, (B) LV2, and (C) LV3. Correlation analysis of differential gut microbiota and differential metabolites between SBP and N‐SBP in (D) LV1, (E) LV2, and (F) LV3. HC, healthy control; LC, liver cirrhosis.

Functional predictions were conducted for the microbiota in LC and SBP. In liver cirrhosis, as Child–Pugh scores increased, there was a notable shift in predominant functionalities toward Human Diseases and Environmental Information Processing at Level 1(Figure 8A). This trend continued at Level 2, with positive correlations observed in functions related to membrane transport, signal transduction, and metabolism, among others. Conversely, functions such as carbohydrate metabolism, replication and repair, and cell motility exhibited negative associations with Child–Pugh scores (Figure 8B). At Level 3, transporters, ABC transporters, and quorum sensing emerged as significant functions positively correlated with Child–Pugh scores, whereas functions related to DNA repair, RNA biogenesis, and metabolism displayed negative correlations (Figure 8C).

In SBP, predominant functionalities associated with human diseases and environmental information processing were positively correlated with SBP, whereas organismal systems exhibited a negative relationship at Level 1 (Figure 8D). At Level 2, positive associations were observed in functions related to membrane transport, drug resistance, and infectious diseases, whereas functions like transport and catabolism showed negative correlations with SBP (Figure 8E). At Level 3, transporters, ABC transporters, and transcription factors displayed positive associations with SBP, whereas functions related to mitochondrial biogenesis and specific metabolic pathways were negatively correlated (Figure 8F).

In conclusion, functional predictions highlight distinct microbial functionalities associated with liver cirrhosis and SBP. Liver cirrhosis is characterized by alterations in various metabolic and signaling pathways, whereas SBP is associated with immune response and infectious disease‐related functionalities. These insights provide valuable information for understanding the pathophysiology and potential therapeutic targets of these conditions.

### 3.6 Correlation analysis of *Ralstonia* , *R. pockettii* , *Faecalibacterium* , *F. prausnitzii* , and liver injury score

**FIGURE 9:**
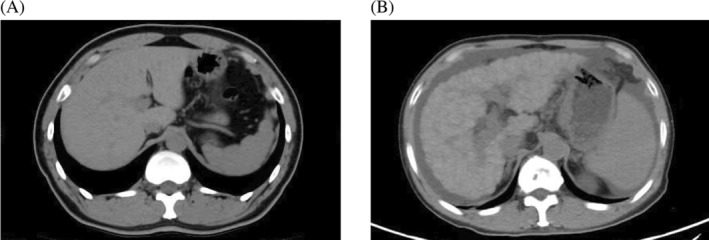
Displays computed tomography (CT) images contrasting healthy individuals (A) with selected cirrhotic patients (B).

We explored the potential association between fecal microbiota composition and IgA levels in patients with varying degrees of liver injury. The microbiota composition in cirrhotic patients was examined alongside high‐resolution CT scans (Figure 9). A cohort of 69 patients underwent routine clinical follow‐up, with the average duration between scanning and fecal microbiological testing being 1 year and 56 days. Linear regression analyses were conducted on various demographic and clinical factors including age, sex, flora, grade of liver injury, and IgA levels of the subjects under investigation. The results of these analyses are summarized in Table 2. Positive correlation between *Ralstonia*, *R. pockettii*, and liver damage score (Child–Pugh), which negative correlation with *Faecalibacterium* and *F. prausnitzii*.

**TABLE 2:** Multi‐factor linear regression analysis.

	Beta	*p*‐value	95%CI	*R* ^2^
Age	0.042	0.224	−0.005, 0.218	
Gender	−0.059	0.855	−3.147, 2.643	
*Ralstonia*	0.546	0.027	0.195, 1.764	0.099
*Ralstonia pockettii*	0.785	0.034	0.008, 1.024	0.098
*Faecalibacterium*	−0.832	0.031	−1.324, −0.057	0.115
*Faecalibacterium prausnitzii*	−1.006	0.048	−2.692, −1.024	0.087

*Note*: Age and gender were included as covariates. Beta coefficient (beta) was bootstrapped to generate 95% confidence intervals (95%CI). Only taxa with *p* < 0.05 and 95% CI that do not span zero are reported.

## 4 DISCUSSION

IgA serves as a linchpin in the intestinal immune response, pivotal for maintaining a delicate balance between host defense and microbial symbiosis.^27^ When bacterial antigens breach the intestinal mucosa, IgA prompts B cells to secrete immunoglobulins, a process modulated by T‐cell dependency.^14^, ^16^ Disruptions in this equilibrium, particularly post recurrent Clostridioides difficile infections, highlight IgA's critical role in gut health. Fecal microbiota transplantation (FMT) has emerged as a promising intervention to restore this balance and alleviate clinical symptoms.^17^ In LC, our investigation found elevated fecal IgA levels, similar to observations in IBD. Notably, IgA levels in LC positively correlated with disease severity assessed by Child–Pugh scores.^19^, ^28^ This suggests IgA's potential utility as a prognostic biomarker in LC progression.

The advent of IgA‐Seq, pioneered by Palm et al., revolutionized bacterial sorting by combining IgA coating with sequencing technology.^29^ Current IBD research posits that IgA‐coated bacteria may harbor pathogenic potential.^28^ Notably, in patients with recurrent Clostridium difficile infections, IgA‐coated bacteria revert to a normal state post symptom resolution,^30^ implicating them in disease etiology.^31^, ^32^ Our study further elucidated IgA's role in LC complications, irrespective of intestinal microflora presence. Thiel et al.^33^ reported a single case of SBP in an IgA‐deficient patient post liver transplantation, suggesting IgA's protective role against SBP. Gupta et al.^34^ suggested that IgA plays a key role in SBP and HSP complications due to its modified immune complex processing. Our data confirms this, emphasizing IgA's importance in maintaining gut immunity and microbial balance, which affects cirrhosis outcomes. IgA's promise as a liver cirrhosis biomarker and its role in disease development point to its therapeutic potential.

16S rRNA gene analyses reveal that higher gut IgA levels correlate with decreased microbial diversity, suggesting that elevated IgA restricts colonization by a wider range of bacterial taxa.^35^, ^36^ It is noteworthy that microbial diversity within the gut microbiota generally aligns positively with an individual's health status.^37^ *Ralstonia*, gram‐negative, nonfermenting bacterium, is commonly found in nature and strongly correlates with host passive immunity. Studies suggest that the acquisition of passive immunity in calves may influence the colonization of *Ralstonia* in their microbiota.^38^ *F. prausnitzii*, a crucial member of the human gut flora, constituting 5%–15% of healthy fecal bacteria, produces butyric acid with anti‐inflammatory effects. It maintains bacterial enzyme activity, protects against intestinal pathogens, preserves intestinal lining integrity, stimulates villi growth, and promotes mucin production, indicating a protective role.^39^ Our study highlights the intricate relationship between chronic liver disease severity and alterations in the fecal microbiota. The Observed Species index showed significant changes between HCs and patients with severe liver disease (Child–Pugh C). The Shannon index revealed a decrease in microbial diversity with worsening liver conditions, indicating a potential loss of beneficial microbial functions that could exacerbate liver disease pathology. β diversity analysis supported the notion of altered microbial communities in liver cirrhosis patients compared to HCs, with distinct clustering patterns in PCoA plots underscoring significant shifts in microbial composition associated with disease progression. At the phylum and genus levels, an increase in IgA‐coated Proteobacteria and a decrease in *Firmicutes* in the LC group compared to the HC group suggest a dysbiosis favoring pathogenic over symbiotic bacteria, which may contribute to inflammatory processes in chronic liver diseases. Specific genera, such as *Ralstonia* and *Streptococcus*, known to be associated with various disease states, may play a role in the pathogenesis of liver cirrhosis. Venn analysis and LEfSe results provide valuable insights into potential biomarkers for liver disease. The identification of specific OTUs unique to each Child–Pugh group and the potential marker bacteria like *F. prausnitzii* and *R. pockettii* in liver cirrhosis patients could pave the way for novel diagnostic and therapeutic strategies.

The study reveals gut microbiota alterations in SBP. Although microbial richness and diversity were similar between N‐SBP and SBP groups, SBP patients showed increased Proteobacteria and decreased Cyanobacteria and *Firmicutes*, potentially contributing to dysbiosis and inflammation. At the genus level, *Pseudomonas* and *Veillonella* were increased, while *Faecalibacterium* was decreased in SBP, aligning with previous findings on gut dysbiosis and inflammatory conditions.

Functional predictions of the microbiota revealed distinct functionalities in liver cirrhosis and SBP. In liver cirrhosis, higher Child–Pugh scores correlated with increased functions related to human diseases, membrane transport, signal transduction, and metabolism, while carbohydrate metabolism, replication and repair, and cell motility decreased. In SBP, there were positive correlations with functions related to human diseases, environmental information processing, membrane transport, drug resistance, and infectious diseases, whereas organismal systems and transport and catabolism functions showed negative correlations. Transporters, ABC transporters, and transcription factors were positively associated with SBP, whereas mitochondrial biogenesis and specific metabolic pathways were negatively correlated.

Comparing these results, both liver cirrhosis and SBP exhibit shifts in microbial functionalities toward human diseases and environmental information processing, albeit with different nuances. Liver cirrhosis shows a more pronounced dysregulation in metabolic and signaling pathways, potentially reflecting the chronic nature of the condition and its impact on various physiological processes. Notably, *Faecalibacterium* and *F. prausnitzii* were identified as shared taxa between SBP and liver cirrhosis, suggesting their involvement in microbial functionalities common to both conditions. SBP shows a distinct pattern linked to immune response and infectious diseases, indicating heightened inflammation and specific microbial dysbiosis. These findings offer valuable insights into gut microbiota alterations in liver cirrhosis and SBP, enhancing our understanding of their pathophysiology. Correlation analysis revealed intriguing associations between specific bacterial taxa and liver injury score (Child–Pugh). *Ralstonia* and *R. pockettii* correlated positively with liver damage score, whereas *Faecalibacterium* and *F. prausnitzii* showed a negative correlation, potentially indicating their roles in liver health.

Our study acknowledges several limitations: the need for a larger, more diverse sample size for robust results, and the inherent limitations of 16S rRNA gene sequencing in providing comprehensive functional information. Establishing causality between gut microbial diversity and liver cirrhosis is challenging in observational studies, requiring future interventional research. Confounding factors such as diet, lifestyle, medication history, and baseline health status influence our findings. Potential functional changes in microbial communities require validation. Future research should validate specific microbe roles in liver health, explore microbial interventions' therapeutic potential, foster interdisciplinary collaborations, and utilize advanced sequencing technologies for deeper insights into microbial community function and structure.

## 5 CONCLUSION

In conclusion, the study underscores the intricate interplay between IgA and the gut microbiota, revealing its significant influence on the pathophysiology of liver cirrhosis and SBP. Elevated IgA levels in liver cirrhosis patients correlate with disease severity, suggesting its potential as a biomarker. The IgA‐Seq technique highlights the role of IgA‐coated bacteria in disease causation, particularly in IBD and Clostridium difficile infections. The study also demonstrates that increased IgA levels are associated with reduced microbial diversity, which may contribute to disease progression. Notable shifts in microbial composition and functionalities between liver cirrhosis and SBP patients indicate distinct patterns of dysbiosis and inflammation. The identification of specific bacterial taxa correlated with liver injury scores offers new insights into the gut‐liver axis and targeted therapeutic interventions. These findings emphasize the importance of maintaining a balanced gut microbiota for liver health and provide a foundation for future research into diagnostic and treatment strategies for liver diseases.

## CONFLICT OF INTEREST STATEMENT

The authors declare no conflict of interest.
